# Lung Function and Health Parameters in Children With and Without Visual Impairment: A Study Protocol for a Two-Phase Study of Comparative Assessment and Randomized Controlled Yoga Intervention

**DOI:** 10.7759/cureus.104085

**Published:** 2026-02-22

**Authors:** Sowjanya Mallempati, Soubhagyalaxmi Mohanty

**Affiliations:** 1 Department of Yoga and Humanities, Swami Vivekananda Yoga Anusandhana Samsthana, Bengaluru, IND

**Keywords:** children, heart rate variability, lung function, physical activity, posture, protocol, randomized controlled trial, visual impairment, yoga

## Abstract

Background

Lung function, a key indicator of physical fitness, is underexplored in children with visual impairment (VI) compared to sighted peers. Although physical activity is known to enhance respiratory function and psychological well-being, children with VI face multiple barriers to participation. This study aims to assess lung function and other health parameters in children with and without VI and to evaluate the effectiveness of yoga in improving these outcomes.

Methodology

The study will be conducted in two phases. Phase one involves a cross-sectional comparative assessment of lung function, autonomic function, muscular performance, postural alignment, and psychological health in children aged 11-18 years with and without VI. Phase two is a randomized controlled trial evaluating the impact of a 12-week yoga intervention on these outcomes in children with VI.

Results

The study is expected to generate comprehensive data on physical, physiological, and psychological health parameters in children with and without visual impairment. It will also evaluate the potential of a 12-week yoga intervention to improve these outcomes in visually impaired children.

Conclusions

This study will provide evidence on health disparities associated with VI in children and assess the role of yoga as a holistic intervention to enhance their overall well-being.

## Introduction

Visual impairment (VI) encompasses the functional limitations of the eye. As per the International Statistical Classification of Diseases 11th Revision (ICD-11), severity is determined by visual acuity in the better eye. Moderate to severe vision impairment is defined by visual acuity ranging from worse than 6/18 to 3/60 inclusive, while blindness is characterized by visual acuity worse than 3/60 [1]. In 2020, it was estimated that around 1.1 billion individuals were living with VI. Projections suggest that by 2050, this number is expected to grow to 1.8 billion people [2]. In India, about 4.95 million are blind (0.36% of the population), and over 35 million have VI (2.55%). VI impacts individuals across all dimensions of life, including physical, emotional, and social domains, thereby reducing overall quality of life [3]. Children with VI often exhibit reduced physical fitness [4], musculoskeletal deformities [5], increased physiological arousal [6], elevated breath rates, and heightened anxiety levels [7]. Sensory impairments, particularly VI, are associated with reduced physical fitness, largely due to compromised mobility that limits participation in physical activities. Although lung function is a key parameter for assessing physical fitness, data on its assessment in children with VI are limited compared to those in the unaffected population.

Research indicates that children and adolescents with low vision and blindness exhibit lower cardiorespiratory fitness and significantly reduced vital capacity levels compared to their sighted peers [8]. Children with VI commonly display increased head tilt, uneven shoulders, thoracic kyphosis, lower lumbar lordosis, and knee deformities [5]. Previous studies have shown that lung function is influenced by factors such as body position, thoracic kyphosis, and sitting posture [9], as well as by physical activity [10], aerobic fitness [11], peripheral muscle strength [12], and depression [13]. However, research on the association between lung function and these factors among individuals with VI is scarce.

Physical activity has been demonstrated to positively affect lipid and lipoprotein levels, body fat, blood pressure, cardiovascular health, self-esteem, anxiety, depression symptoms, and academic performance in children and adolescents [14]. Participation in different physical activities, such as climbing, trampoline jumping, and swimming, significantly influences the functional respiratory values for visually impaired and blind children. Children and adolescents with VI who do not engage in sports tend to exhibit substantially lower values of forced vital capacity (FVC), forced expiratory volume in one second (FEV1), and maximal voluntary ventilation (MVV) compared to standard or predicted values. Conversely, children with VI involved in performance sports demonstrate significantly elevated levels of FVC, FEV1, and peak expiratory flow (PEF) compared to standard values [15].

Despite the importance of physical activity in promoting both physical and psychological well-being among children with disabilities [4], several perceived barriers hinder their participation. These barriers include inadequate parental support, limited opportunities, fear of injury, absence of trained physical educators, insufficient security measures, motivation issues, inadequate professional training and information about available activities, and lack of self-efficacy [16]. Introducing various forms of physical activity into practice in developing countries is challenging due to the complexity of creating cost-effective programs that can be widely disseminated, adopted, and implemented. In this context, yoga, an ancient, widely accepted, easily accessible, and cost-effective mind-body technique, emerges as a favorable option. Once mastered, yoga can be practiced independently by visually impaired individuals, unlike performance sports, which often require ongoing assistance. This study aims to assess lung function in children with and without VI, explore its relationship with physical and psychological health parameters, and evaluate the effectiveness of yoga in improving these outcomes.

## Materials and methods

Study design and setting

**Figure 1:**
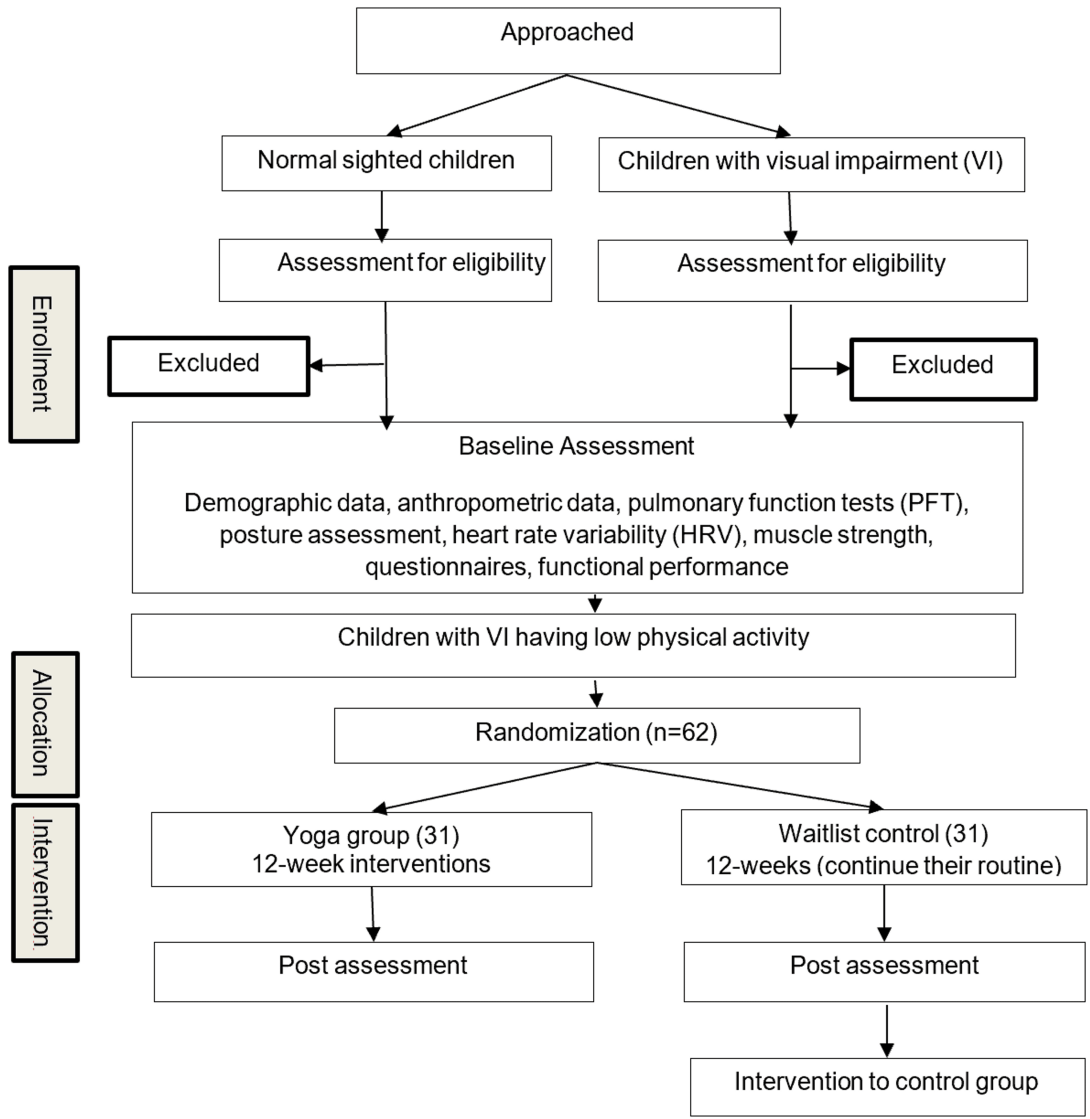
Trial flowchart.

The present study has two phases. Phase one involves a comparative assessment between children with VI and normally sighted children. Phase two is a two-group parallel, randomized waitlist-controlled trial with a 1:1 allocation ratio to investigate the impact of a 12-week yoga intervention on lung function and other factors among children with VI (Figure 1).

This manuscript adheres to the Standard Protocol Items: Recommendations for Interventional Trials (SPIRIT) 2013 guidelines, and the randomized controlled trial (RCT) study will be conducted and reported in accordance with the requirements of the Consolidated Standards of Reporting Trials (CONSORT) statement [17]. Children with VI will be recruited from specialized schools such as Devnar School for the Blind in Hyderabad, Telangana, India. At the same time, children with normal vision will be selected from regular schools. Participants from the phase one study who meet the study criteria will be included in the RCT (phase two).

Objectives of the study

(1) To compare lung function parameters (FVC, FEV₁, and peak expiratory flow rate (PEFR)) between children with VI and normal-sighted children. (2) To compare physical activity, posture, muscle strength, autonomic function, and psychological parameters between children with VI and normal-sighted children. (3) To examine the association between lung function and physical, physiological, and psychological parameters among children with VI. (4) To evaluate the effect of the yoga intervention on lung function, posture, muscle strength, autonomic function, and psychological parameters in children with visual impairment.

Eligibility criteria

The study group will be selected based on the inclusion and exclusion criteria detailed in Table 1.

**Table 1:** Inclusion and exclusion criteria. PAQ-A = Physical Activity Questionnaire for Adolescents

	Inclusion criteria	Exclusion criteria
Phase one	Children aged 11-18 years of both genders	Children with any chronic disease
	Children with moderate to severe visual impairment or total blindness	Multiple impairments
	Age-matched normally sighted children	Parents unwilling to provide informed consent
	Being able to understand English	Subjects under any long-term medications
Phase two	Physically low active (mean of total items of PAQ-A <2)	Any injury restricts the practice of yoga.
	-	Exposure to yoga or any exercise six months before the study commenced

Intervention

The validated yoga module customized for children with VI to improve their lung function will be administered to the intervention group. The yoga sessions mainly include asanas, sun salutations, specialized breathing techniques, and relaxation techniques. The intervention will be conducted for one hour per day, six days a week, spanning a duration of 12 weeks. To accommodate the unique learning needs of children with VI, the intervention will be provided to a group of 15-16 subjects at a time. Throughout the study, the same yoga intervention will be given to the yoga group by utilizing appropriate teaching models tailored for them [18]. A certified and experienced yoga instructor will conduct the sessions during the evening hours, following the conclusion of their school day. Daily attendance will be logged, and the absentees will be personally contacted to inquire about their absence. To promote adherence, the trainer will allocate personal time to each participant to address their concerns and provide motivation throughout the intervention phase. To ensure participants’ comfort and provide access to necessities such as water and toilets, as well as address mobility challenges, classes will be held in a hall within the school premises. We opted for a waitlist design for the inactive control group to ethically provide care to them. During recruitment, they will be instructed to continue their usual daily routines. Post-assessment, they will be offered the same yoga intervention as the intervention group. The detailed description of interventions is provided in Table 2.

**Table 2:** Yoga intervention protocol.

Name of the practice	Number of rounds	Duration
Opening prayer with chanting om	1 round	1 minute
Breathing practices		
Hand in and out breathing	5 rounds	30 seconds
Hand stretch breathing	3 rounds each	2 minutes
Shashankasana breathing	7 rounds	1 minute
Tiger breathing	7 rounds	1 minute
Loosening practices		
Neck movements	5 rounds	90 seconds
Forward-backward bending	5 rounds	1 minute
Suryanamaskar	12 rounds	19 minutes
Savasana	1 round	1 minute
Asanas		
Trikonasana	1 round	1 minute
Vakrasana	1 round	1 minute
Ustrasana	1 round	1 minute
Gomukhasana	1 round	1 minute
Setubandhasana	1 round	1 minute
Matsyasana	1 round	1 minute
Dhanurasana	1 round	1 minute
Bhujangasana	1 round	1 minute
Makarasana	1 round	1 minute
Pranayama		
Kapalabhati	2 rounds	2 minutes
Bhastirka	2 rounds	2 minutes
Sectional breathing	5 rounds each	4 minutes
Nadi shudhi	21 rounds	4 minutes
Bhramari	18 rounds	3 minutes
Om chanting	-	2 minutes
Deep relaxation technique	1 round	5 minutes
Ending prayer	1 round	1 minutes

Outcome measurements

Demographic details include factors such as age, gender, education level, degree or percentage of VI, parents’ consanguineous marriage, and socioeconomic status (SES). Anthropometric details comprise measurements such as height, weight, and body mass index (BMI).

Primary Outcome

Throughout both phases of the study, lung function parameters FVC, FEV₁, and PEFR will serve as the primary outcomes for group comparisons.

Secondary Outcome

Physical activity, posture, muscle strength, autonomic function (heart rate variability), psychological parameters, and overall physical fitness will be considered secondary outcomes.

Phase one

Barriers to physical activity among children with VI will be assessed using the Physical Activity Barriers Questionnaire for Children With Visual Impairments (PABQ-VI). General physical activity levels will be determined using the Physical Activity Questionnaire for Adolescents (PAQ-A). Outcome measures will also include pulmonary function tests (PFTs) using a spirometer to evaluate lung function, objective assessment of posture using the Posture Screen mobile application, heart rate variability will be evaluated using a portable single-channel ECG module, and upper extremity muscle strength will be assessed using the Lafayette manual muscle test. Depression levels will be gauged using the Children's Depression Inventory (CDI)-2 questionnaire. All variables will be compared between children with VI and those with normal sight, exploring relationships between lung function and factors such as physical activity, posture, heart rate variability, muscle strength, and psychological parameters in children with VI. The schedule for enrollment, intervention, and assessments, following the SPIRIT 2013 guidelines, has been detailed in Table 3 and Table 4.

**Table 3:** Phase one trial schedule. VI = visually impaired; PA = physical activity; T0 = enrollment; T1 = baseline assessment of visually impaired children; T2 = baseline assessment of normal-sighted children

Study time points	Enrollment	Baseline assessment	
	T0	T1	T2
		Children with VI	Normal-sighted children
Eligibility screening	X	-	-
Informed consent	X	-	-
Assessments			
Demographic details	-	X	X
Anthropometric details		X	X
Physical activity levels		X	X
Lung function		X	X
Posture assessment		X	X
Heart rate variability		X	X
Upper extremity muscle strength		X	X
Depression		X	X
Barriers to PA		X	-
Functional performance		X	-

**Table 4:** Phase two trial schedule. T0 = enrollment of children with visual impairment from phase one; T1 = intervention to the yoga group for three months; T2 = post-assessment of both groups; T3 = intervention to the control group

Phase two				
Study time points	Enrollment	Intervention	Post-assessment	Intervention
	T0	T1	T2	T3
		Yoga group	Yoga and control group	Control group
Eligibility screening	X	-	-	-
Intervention		X	-	X
Assessments	-	-	-	-
Anthropometric details			X	
Lung function			X	
Posture assessment			X	
Heart rate variability			X	
Upper extremity muscle strength			X	
Depression			X	
Functional performance			X	

Phase two

The outcome measures for the RCT study will encompass lung function, posture assessment, heart rate variability, upper extremity muscle strength, and depression, utilizing the same tools employed before and after 12 weeks of intervention. Functional performance will be evaluated using the 6-Minute Walk Test (6-MWT).

Sample size

Based on an earlier study, the sample size was calculated using GPower software based on PEFR data, with an alpha of 0.05, power of 0.80, and an effect size of 0.8. A 20% attrition rate was incorporated to account for potential dropouts during the three-month yoga intervention in children with VI, who may experience health or participation-related challenges [19].

Recruitment

Participants will be identified by reaching out to special school administrators and teachers. An announcement will be made in the schools to raise awareness about the study and encourage participation. Additionally, teachers and supporting staff will be encouraged to spread the word within school networks. Parents or guardians will also receive information about the study orally during parent-teacher conferences. Recruitment and enrollment of participants will be performed by the study investigator.

Randomization and blinding

In phase two, participants will be randomly allocated to either the control or experimental group in a 1:1 ratio. Allocation concealment will be ensured by an independent researcher using sequentially numbered, opaque, sealed envelopes prepared according to the RStudio-generated randomization sequence. The study investigator will enroll participants and open the envelopes only after enrollment, revealing the group assignment. Considering the nature of the intervention and the participants being special children, the study will be conducted as an open trial. Due to logistical constraints, blinding the participants to allocation is not feasible. Although outcome assessors will not be informed of group allocation, complete blinding cannot be guaranteed as this information may be disclosed by school staff or the children themselves. However, the data analyst will be blinded to the study groups by using code identification for the two groups.

Data collection method

Sociodemographic data will be collected via a questionnaire. Data assessors will be trained on proper data collection procedures and ethical guidelines before starting the study. The principal investigator will supervise the data collection process to ensure accuracy, consistency, and data quality. Assessors will consult the principal investigator for any questions or uncertainties that arise during data collection. Trained research assistants will administer the noninvasive tests by following the standard procedure. The detailed information about instruments and the method of assessment is presented in Table 5. Data will be monitored by the principal investigator. The yoga instructor will be instructed to prioritize participant safety, taking care to prevent any falls or injuries during the intervention. Participants will be advised to promptly inform the study coordinator of any adverse events experienced throughout the study. Additionally, after each session, participants will have a personal meeting with the instructor to address any concerns and minimize potential harm.

**Table 5:** Summary of variables and assessment procedures. FVC = forced vital capacity; FEV1 = forced expiratory volume in one second; PEFR = peak expiratory flow rate; ECG = electrocardiogram

Variable	Procedure for assessment
Lung function	Pulmonary function test will be conducted using an NDD Easy on-PC spirometer, following the standards established by the American Thoracic Society/European Respiratory Society for lung function testing. The test will include standard spirometry measures such as FVC, FEV1, FEV1/FVC ratio, and PEFR, among others. The technician will provide a demonstration on how to perform the test and offer clear instructions throughout the procedure. Three reproducible manoeuvres will be obtained in a sitting position, and the highest values will be documented and used for analysis [20]
Heart rate variability	The ECG signal will be recorded using a portable single-channel ECG module. This module, of the plug-and-play variety, can record any one of the bipolar leads (Lead 1, 2, 3) ECG at a rate of 8,000 samples per second. The RR interval obtained from the portable HRV module will be as reliable as the gold standard Biopac MP36 [21]. Following 10 minutes of rest in a supine posture, limb lead 2 ECG will be recorded for 10 minutes in a noise-free environment
Isometric muscle strength	The Lafayette manual muscle tester system (Model 01165, Lafayette Instrument Company, Indiana, USA) will be used to objectively quantify isometric muscle strength. Standardized dynamometer placements and test measurement procedures will be employed to acquire data for shoulder internal rotation and shoulder external rotation. Three successive trials will be conducted with a 10-second rest between each trial in a sitting position. The maximum value obtained will be selected for analysis. The Lafayette hand-held dynamometer demonstrates good to excellent reliability and validity for measuring isometric muscle strength [22]
General physical activity levels	The general physical activity levels will be assessed using the Physical Activity Questionnaire for Adolescents, a self-administered 7-day recall tool designed to evaluate the extent of moderate to vigorous physical activity performed. The reliability and validity of the tool are good and are sensitive to gender differences [23]. This is one of the tools widely used among adolescents and visually impaired adolescents to assess physical activity
Posture	The Posture Screen mobile application will be used to assess posture during the standing position objectively. This application demonstrates strong rater reliability and has shown preliminary evidence of construct validity [24]
Depression symptoms	Depression will be assessed using the 28-item Children’s Depression Inventory-2 questionnaire. Each item provides three response options: 0 for the absence of symptoms, 1 for mild or probable symptoms, and 2 for definite symptoms. A higher score indicates a higher level of depressive symptoms. It is a reliable and well-validated tool for assessing depressive symptoms [25]
Barriers to physical activity	The Physical Activity Barrier Questionnaire-Visually Impaired (PABQ‑VI) short version will be utilized to assess barriers to physical activity among individuals with visual impairments. The PABQ‑VI is a reliable and valid tool for assessing personal, social, and environmental barriers to physical activity in subjects with visual impairments [26]
Functional performance	Functional performance will be evaluated using the 6-minute walk test, conducted in accordance with the standards outlined by the American Thoracic Society (ATS Statement: Guidelines for the Six-Minute Walk Test) [27]
Demographic and physical health assessment	Socioeconomic status will be assessed using the modified Kuppuswamy socioeconomic scale updated for 2021. It classifies families into 5 groups: upper class, upper middle class, lower middle class, upper lower, and lower socioeconomic class [28]. Information regarding consanguineous marriage among children’s parents and the degree of visual impairment and age of the children will be collected from school records. Height will be measured using a stadiometer, while body weight will be measured using a digital scale. Body mass index will be calculated as weight (kg) divided by height squared (m²)

Ethics and dissemination

The study has received approval from the Institutional Ethics Committee at Swami Vivekananda Yoga Anusandhana Samsthana (SVYASA) in Bangalore, India (IEC number: RES/IEC-SVYASA/277/2022). Any changes to the study plan will be communicated to the Ethics Committee before being put into action. Additionally, the study has been registered with the Clinical Trials Registry - India (CTRI) (registration number: CTRI/2023/07/055165). Study coordinators contacted multiple schools and acquired permission letters from those interested in participating. The principal investigator will orally explain the study procedure and the level of involvement expected from children to their parents. Then, informed consent will be obtained from the parents of children who meet the study criteria. Children will be free to withdraw from the study at any time. The research staff will administer the questionnaires to the subjects, and these responses will be written down on hard copy and later transferred to an Excel spreadsheet. All study-related information will be securely stored in a locker at the study site. During the study, only research staff will have access to the personal details of the subjects. The final database will be accessible solely to the principal investigator, study coordinator, and statistician. The study results will be shared through publication in open-access, peer-reviewed journals, presentations at conferences, and local seminars.

## Results

Descriptive statistics will be used to summarise participants’ sociodemographic details at baseline. Categorical data will be analysed using the chi-square test to determine any significant associations. The normality of the data will be assessed using the Shapiro-Wilk test, while Levene’s test will be applied to ensure the equivalence of variances. To compare outcomes between the VI and normal-sighted groups, either an independent samples t-test or a Wilcoxon rank-sum test will be employed, depending upon the normality of the data. Correlational analysis will be conducted to identify associations between lung function and other outcomes. In phase two, the intervention group will be compared against the control group for all primary and secondary outcomes. We will choose the appropriate parametric or non-parametric test based on the normality of the data. The missing data will be substituted using the predictive mean matching (PMM) imputation method. Analysis will be performed using SPSS version 21 (IBM Corp., Armonk, NY, USA) or RStudio. The study is registered with CTRI and has received Ethics Committee approval in mid-calendar year 2023.

## Discussion

Yoga as an alternative therapy has shown promise in enhancing physical fitness, increasing PEFR value [19], normalizing breath rate [6], and reducing fear of falling among children with VI [29]. Additionally, previous studies have shown that yoga and corrective exercises can help improve hyperkyphosis in elderly individuals [30]. However, research on the effects of yoga specifically on lung function in children with VI remains limited. To our knowledge, this study represents a pioneering effort with a robust design aimed at investigating the lung function and various factors such as physical activity, posture, heart rate variability, muscle strength, and psychological parameters in children with and without VI. Additionally, it seeks to explore the potential effects of a yoga intervention on these parameters. Through a waitlist-controlled design, we ethically provide all participants access to the yoga intervention. The findings may support yoga as a feasible strategy to enhance overall health in children with VI. This allows us to assess its impact on lung function, physical activity, posture, heart rate variability, muscle strength, and psychological well-being.

Strengths and limitations

This study combines objective health measures, such as lung function, muscle strength, posture, and heart rate variability, with validated psychological assessments, providing a comprehensive evaluation of children’s health. The RCT design, including a waitlist control group, allows all participants to benefit from the yoga program while providing a rigorous and ethically responsible evaluation of its effects. Inclusion of both visually impaired and sighted children allows meaningful comparisons and identification of specific needs in the VI population. Additionally, the structured 12-week yoga intervention with standardized protocols supports reproducibility and potential scalability for rehabilitation programs. Despite the robust design, several limitations should be acknowledged. Key limitations of the study include the open trial design, which may introduce performance and reporting bias as participants are aware of group allocation and may respond in socially desirable ways, and observer bias as assessors cannot be fully blinded. Variations in social support among participants are not controlled for, which could influence outcomes. Dropouts and missing data may also affect internal validity, though strategies such as imputation are planned to minimize their impact. Although participants come from different regions of the state, the study is conducted at a single center, which may limit generalizability to other regions.

## Conclusions

This study aims to provide comprehensive insights into the lung function, physical fitness, posture, autonomic function, muscle strength, and psychological well-being of children with and without VI. It is designed to evaluate the effects of a 12-week yoga intervention on these outcomes among children with VI. The study is hypothesis-driven and will examine whether yoga-based intervention is feasible and associated with improvements in health-related outcomes. The results are expected to contribute to the existing evidence base and may help inform future rehabilitation research and program development for children with VI.
